# Identification of diagnostic biomarkers and potential therapeutic targets for biliary atresia via WGCNA and machine learning methods

**DOI:** 10.3389/fped.2024.1339925

**Published:** 2024-06-26

**Authors:** Lei Xu, Ting Xiao, Biao Zou, Zhihui Rong, Wei Yao

**Affiliations:** ^1^ Department of Pediatrics, Tongji Hospital, Tongji Medical College, Huazhong University of Science and Technology, Wuhan, Hubei, China; ^2^ Department of Ultrasonography, Tongji Hospital, Tongji Medical College, Huazhong University of Science and Technology, Wuhan, Hubei, China; ^3^ Department of Oncology, Tongji Hospital, Tongji Medical College, Huazhong University of Science and Technology, Wuhan, Hubei, China

**Keywords:** biliary atresia, bioinformatics, machine learning, therapeutic targets, biomarkers

## Abstract

Biliary atresia (BA) is a severe and progressive biliary obstructive disease in infants that requires early diagnosis and new therapeutic targets. This study employed bioinformatics methods to identify diagnostic biomarkers and potential therapeutic targets for BA. Our analysis of mRNA expression from Gene Expression Omnibus datasets revealed 3,273 differentially expressed genes between patients with BA and those without BA (nBA). Weighted gene coexpression network analysis determined that the turquoise gene coexpression module, consisting of 298 genes, is predominantly associated with BA. The machine learning method then filtered out the top 2 important genes, *CXCL8* and *TMSB10,* from the turquoise module. The area under receiver operating characteristic curves for *TMSB10* and *CXCL8* were 0.961 and 0.927 in the training group and 0.819 and 0.791 in the testing group, which indicated a high diagnostic value. Besides, combining *TMSB10* and *CXCL8*, a nomogram with better diagnostic performance was built for clinical translation. Several studies have highlighted the potential of *CXCL8* as a therapeutic target for BA, while *TMSB10* has been shown to regulate cell polarity, which was related to BA progression. Our analysis with qRT PCR and immunohistochemistry also confirmed the upregulation of *TMSB10* at mRNA and protein levels in BA liver samples. These findings highlight the sensitivity of *CXCL8* and *TMSB10* as diagnostic biomarkers and their potential as therapeutic targets for BA.

## Introduction

Biliary atresia (BA) is a severe infantile biliary obstructive disease characterized by progressive inflammation and fibrosis, with a global annual incidence of 1 in 8,000–18,000 newborns (1, 2). Without surgery, BA can rapidly progress to end-stage liver cirrhosis within two years, making it the leading cause of pediatric liver transplants (3). Kasai portoenterostomy, a surgical procedure that restores bile flow within 30 days of life, could slow disease progression and prevent the need for liver transplants (4, 5). However, BA is typically diagnosed at an average age of 40–50 days, making it challenging to confirm it in its early stages (6, 7).

The gold standard for diagnosing BA is intraoperative cholangiography, an invasive, time-consuming, and costly procedure associated with radiation damage. No clinical, laboratory, or imaging feature currently allows for the reliable diagnosis of BA before operation. Among traditional diagnostic methods, liver biopsy is the most reliable method of pre-laparotomy diagnosis, with reported diagnostic accuracy ranging from 88.2% to 96.9% (8, 9). Nevertheless, some histopathological characteristics of biliary atresia may considerably overlap with those of nonobstructive etiologies of infantile cholestasis. Improving diagnostic accuracy would avoid unnecessary diagnostic procedures for a definitive and early diagnosis. Additionally, despite successful Kasai portoenterostomy, over 75% of patients with BA eventually require liver transplantation due to their growing hepatic impairment. Identifying new therapeutic targets may delay progressive hepatic dysfunction and decrease the incidence of liver transplantation.

RNA sequencing (RNA-seq) technology has made substantial advancements over the past several years and has emerged as a crucial technique for discovering new diagnostic biomarkers and therapeutic targets (10). Profiling the RNA expression patterns of BA samples can yield valuable insights into novel biomarkers that can improve diagnostic accuracy and therapeutic strategies.

We employed Gene Expression Omnibus (GEO) datasets to obtain mRNA expression profiles of BA liver biopsy samples and identify the hub gene related to BA. First, we identified differentially expressed genes (DEGs) between patients of BA and nBA. Second, the DEGs were then used to determine the gene coexpression module mostly associated with BA using weighted gene coexpression network analysis (WGCNA) which is a powerful bioinformatics method for analyzing gene association patterns and connecting clinical traits to gene coexpression modules (11–13). Third, we identified *CXCL8* and *TMSB10* as the most crucial genes for BA from the gene coexpression module through machine learning techniques. Finally, we further validated the diagnostic potential of *TMSB10* and *CXCL8*. To facilitate clinical translation, we developed a nomogram that integrates *TMSB10* and *CXCL8* to enhance the accuracy of BA diagnosis.

## Materials and methods

### Dataset collection

The mRNA expression profiles of the training group (GSE46995) were acquired from the GEO database and then were normalized and standardized to ensure sample comparability. The training group included 64 patients with BA and 21 nBA (normal and intrahepatic Cholestasis). Datasets GSE122340 (171 BA and 7 normal), GSE221346 (8 BA and 10 intrahepatic cholestasis) and GSE206364 (9 normal and 10 intrahepatic cholestasis) were downloaded from the GEO database and then merged into one group, which was further defined as the testing group (179 BA and 36 nBA).

### Differential expression gene analysis

DEGs between patients of BA and nBA were identified from the mRNA expression profiles of the training group using the “limma” R package (version 3.52.1). Wilcoxon rank-sum test was used to verify the mRNA expression difference of each gene between the BA and the nBA group, and the *P *< 0.05 and | Log (fold change) | >0 were chosen as the threshold for defining DEGs.

### Gene coexpression module construction

Gene coexpression modules were constructed with DEGs using the “WGCNA” R package (version: 1.70-3). The key steps for creating coexpression gene modules using the WGCNA were as follows: Firstly, an adjacency matrix, representing the correlation coefficient matrix between genes, was established. Secondly, a gene topological overlap matrix (TOM) was generated based on the adjacency matrix. Thirdly, the hierarchical clustering tree was produced using hierarchical clustering for TOM-based dissimilarity (dissTOM), and the dynamic tree cut method was used to identify the gene coexpression modules from this tree. Finally, the relationship between gene coexpression modules and clinical characteristics was ascertained through Pearson's correlation analysis, selecting the most strongly correlated coexpressed gene module for further investigation.

### Identification of hub gene via machine learning

The DEGs from the key gene modules were employed as explanatory factors, while the diagnosis of BA or not was the response variable. The four machine learning algorithms, support vector machine (SVM) (11), eXtreme Gradient Boosting (XGB) (12), generalized linear models (GLM) (13), and random forest (RF) (14, 15) were constructed using the“xgboost” (version 1.7.5.1), “kernlab” (version 0.9-31), and “randomForest” (version 4.7-1.1) R packages. To ensure result comparability, all models were built using default parameters. Subsequently, the “DALEX” (version 2.4.2) R package was used to evaluate the machine learning models and determine the residual distribution of each model. The model with the lowest residual distribution was selected as the most appropriate. Finally, the feature importance of the genes for the most appropriate model was assessed via the permutation importance, and the top important genes with the highest root mean square error have the greatest impact on clinical features and were selected as the hub genes for further study.

### Nomogram construction and validation

Using “rms” (version: 6.3-0) R packages, our study developed a nomogram based on gene biomarkers to predict BA via the logistic regression model. The diagnostic performance of the nomogram was estimated using the ROC curve and C-index. Additionally, calibration curves were employed to assess the accuracy between the observed and predicted rates. The utility of the nomogram for decision-making was evaluated through decision curve analysis (DCA) using the “rdma” (version: 1.6) R packages.

### Investigation of immune characteristics related to BA

Based on gene expression profiles, CIBERSORT (16), a flexible computational algorithm, was used for quantifying the proportions of 22 types of immunocyte subclusters for each patient with BA and nBA.

### Quantitative real-time PCR

Quantitative real-time PCR (qRT‒PCR) were performed to detect mRNA expression of *TMSB10*. Total RNA were separated by TRIzol reagent (Life Technologies,CA) and was then convertted into complementary DNA (cDNA) via a reverse transcriptase kit (Takara Bio Inc., Dalian, China) according to the manufacturer's instructions. cDNA was used to perform qRT-PCR via the SYBR Premix EX Taq kit (Takara Bio Inc., Dalian, China) according to the standard protocol. The primer listed in Table 1 were obtained from PrimerBank (pga.mgh.harvard.edu/primerbank).

**Table 1:** The primers for target genes.

Gene	Forward primer	Reverse primer
TMSB10	GAAATCGCCAGCTTCGATAAGG	TCAATGGTCTCTTTGGTCGGC
B-ACTIN	CAGATGTGGATCAGCAAGCAGGAG	AAGCCATGCCAATGAGACTGAGAAG

### Immunohistochemical staining

Immunohistochemical (IHC) staining were performed to detect protein expression of *TMSB10* using the standard streptavidin–biotin peroxidase complex (SABC) method. In short, liver specimens were fixed in 10% neutral formalin, embedded in paraffin blocks and sliced into 4 um sections. The sections were dewaxed and rehydrated in graded ethanol concentrations. After being washed in distilled water, the sections were boiled in sodium citated buffer for epitope retrieval, treated with 3% hydrogen peroxide to inhibit endogenous peroxidase and inclubated with 10% normal serum to block nonsepecific antibody binding. Next, the sections were successively incubated with primary antibody overnight at 4°C and secondary antibody for 30 min at room temperature. Finally, after been counterstaineed with 3,3N-Diaminobenzidine Tertrahydrochloride and hematoxylin, the sections were dehydrated, coverslipped, and observed with microscope. Base on imageJ software and “IHC Toolbox” plug-in, the staining intensity were evaluated via avarege optical density (AOD). The primary and secondary antibodies are listed in Table 2. Clinical information on patients for RT-PCR and immunohistochemistry were listed in Supplementary Table S1.

**Table 2:** The antibody information of the target gene.

Gene	Primary antibody	Secondary antibody
TMSB10	YT6361 (ImmunoWay), 1/200 dilution	HRP Goat anti-rabbit IgG H&L (abs20002), 1/500 dilution

## Results

### Identification of DEGs between BA and nBA

**Figure 1:**
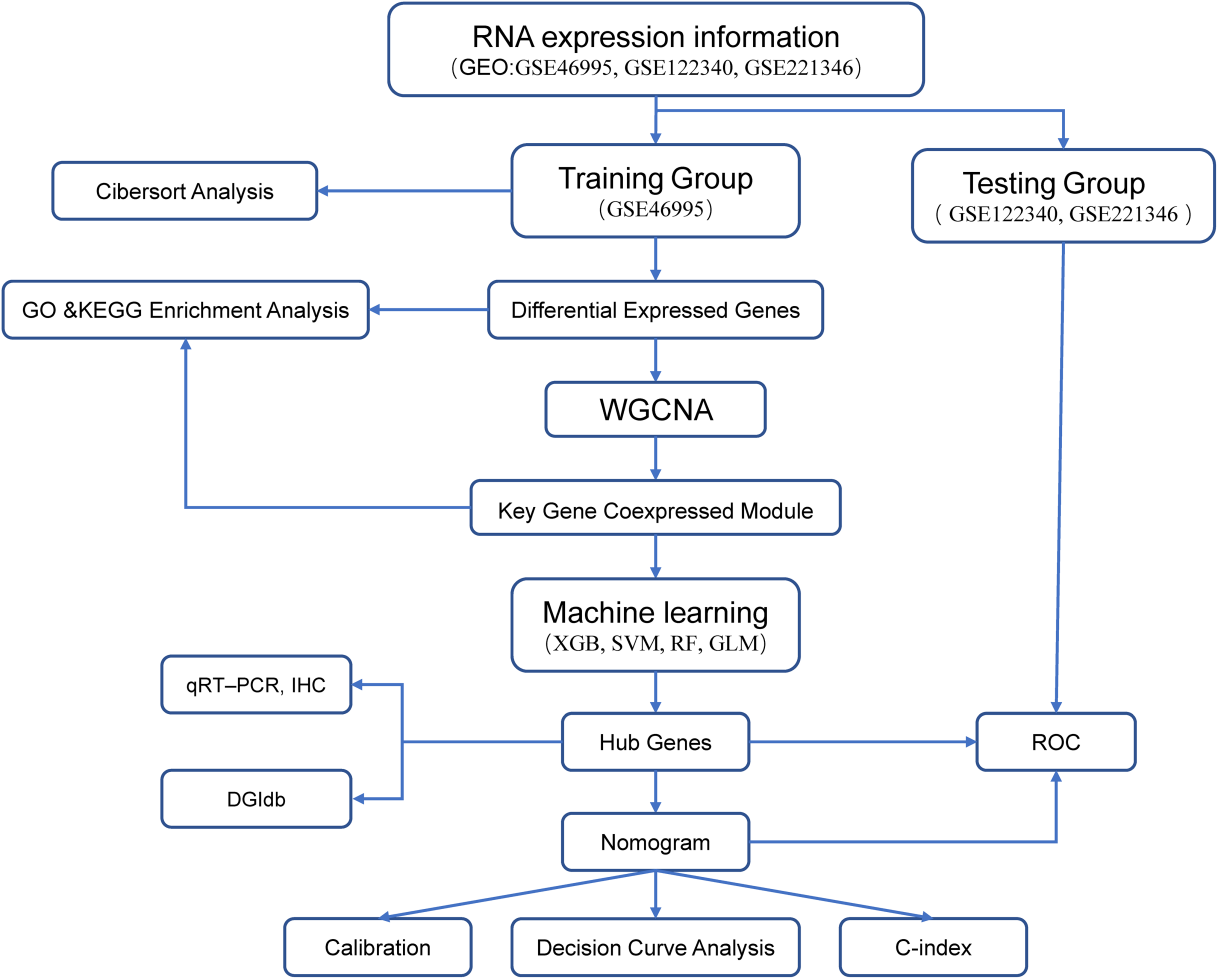
Analysis flowchart of our study.

**Figure 2:**
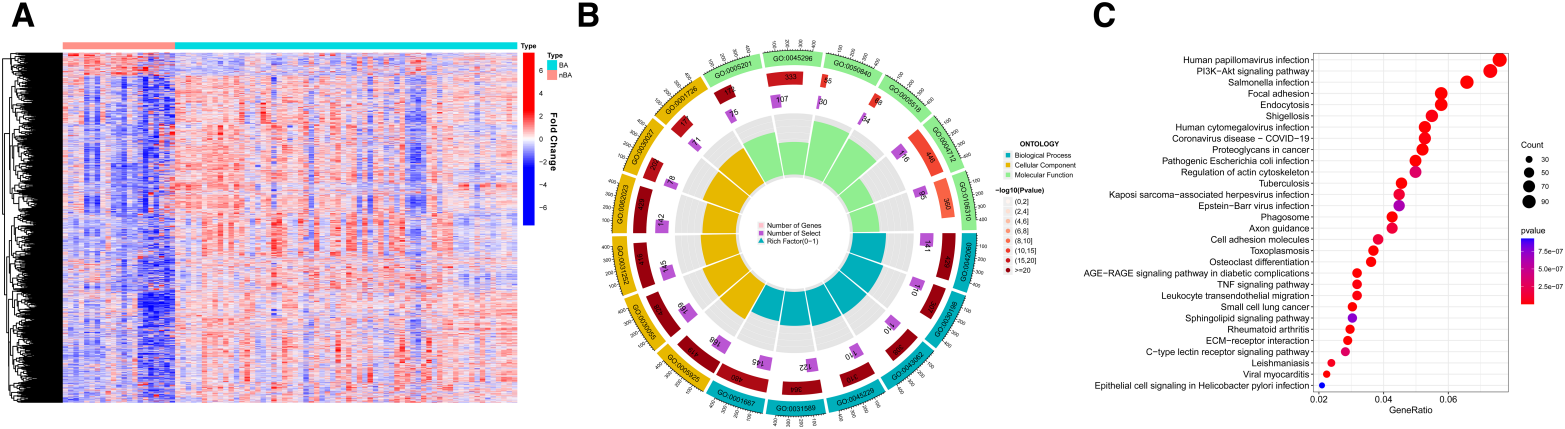
The differentially expressed genes (DEGs) between BA and nBA. (**A**) The heatmap shows the training group's DEGs between BA and nBA. (**B**) GO enrichment analysis for DEGs. (**C**) KEGG enrichment analysis for DEGs.

The analysis flowchart is shown in Figure 1. We analyzed the mRNA expression profiles of 64 BA and 21 nBA samples, identifying 3,273 DEGs between them. Among them, 216 genes were downregulated in BA, while 3,057 were upregulated (Figure 2A; Supplementary Table S2). The 3,273 DEGs underwent enrichment analysis to explore the biological processes involved. GO enrichment analyses revealed that the top three biological processes were ameboidal-type cell migration, wound healing, and cell-substrate adhesion. The top three cellular components included cell-substrate junction, focal adhesion, and cell leading edge, while the top three molecular functions were GTPase regulator activity, nucleoside-triphosphatase regulator activity, and protein serine/threonine/tyrosine kinase activity (Figure 2B; Supplementary Table S3). KEGG pathway analysis also highlighted the top five pathways: Human papillomavirus infection, PI3K-Akt signaling pathway, Salmonella infection, Focal adhesion, and Endocytosis (Figure 2C; Supplementary Table S4).

### WGCNA identified key gene coexpression modules related to Ba

**Figure 3:**
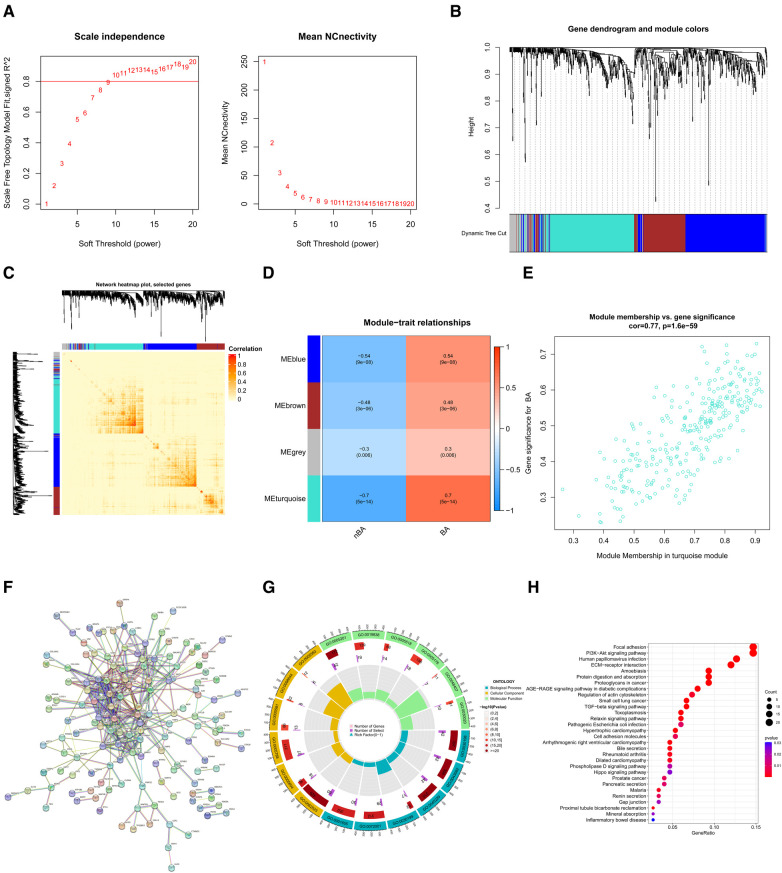
Identification of key gene coexpression modules related to BA using WGCNA. (**A**) Soft thresholding power (β) was determined as 9 for WAGCA when the correlation coefficient was set to 0.8. (**B**) Clustering dendrograms determined four gene coexpression modules using the DEGs. (**C**) The network heatmap plot shows the correlation between genes in four modules; darker red indicates a weaker correlation, and light red indicates a greater significant correlation. (**D**) The heatmap displays the correlation between the coexpression modules and BA and demonstrates that the turquoise module (R = 0.7, *P* = 5e−14) best matched BA. (**E**) The scatter plot shows the correlation between module membership and gene significance in the turquoise module. (**F**) The PPI network visualized interaction among genes in the turquoise module. (**G**) GO enrichment analysis for genes in the turquoise module. (**H**) KEGG enrichment analysis for genes in the turquoise module.

Based on the WGCNA method, we used the 3,273 DEGs to construct gene coexpression modules. The selection of an optimal soft thresholding power of 9 ensured that the gene coexpression network was scale-free (Figure 3A). As a result, four gene coexpression modules emerged: the grey module (64 genes), brown module (171 genes), blue module (289 genes), and turquoise module (298 genes) (Figure 3B). DissTOM was employed to visualize the correlations between the DEGs (Figure 3C).

We calculated the correlations between BA and these four modules and demonstrated that the turquoise module (*R* = 0.7, *P* = 5e−14) exhibited the strongest association with BA (Figure 3D). Besides, the 298 genes in the turquoise module had a highly positive correlation with BA (*R* = 0.77, *P* = 1.6e−59) (Figure 3E). A protein-protein interactions (PPI) network, constructed through the STRING database, visualized interactions among these 298 genes (Figure 3F). The biological processes of the genes in the turquoise module were further investigated via enrichment analyses. The GO enrichment analyses indicated that the top three biological processes were the extracellular matrix (ECM) organization, ECM organization, and external encapsulating structure. The top three cellular components included collagen-containing ECM, endoplasmic reticulum lumen, and apical part of the cell. The top three molecular functions were ECM structural constituent, growth factor binding, and glycosaminoglycan binding (Figure 3G). The top five enriched KEGG pathways included the Focal adhesion, PI3K-Akt signaling pathway, human papillomavirus infection, ECM-receptor interaction, and amoebiasis. (Figure 3H).

### Identification of hub genes related to BA via machine learning

**Figure 4:**
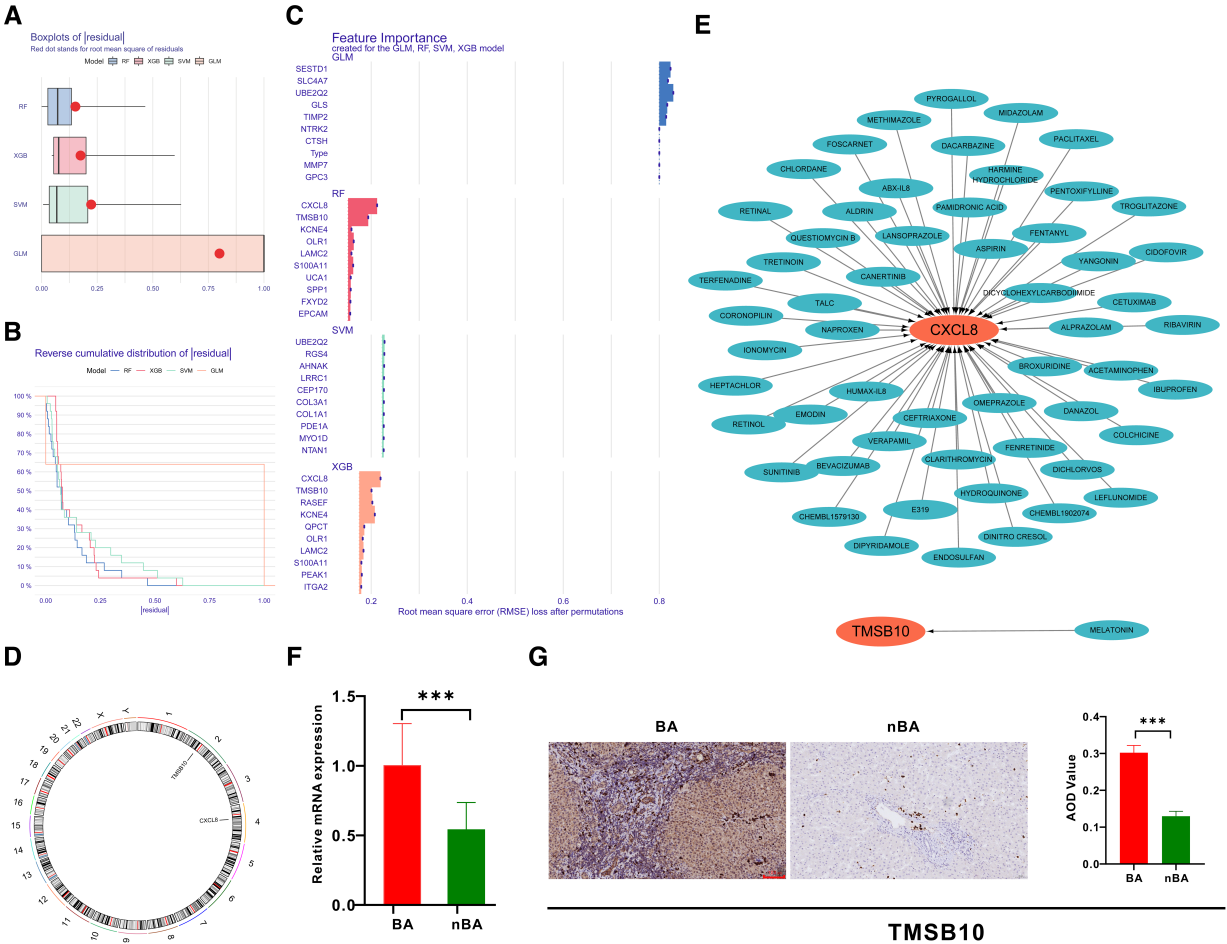
Identification of hub genes related to BA using machine learning. (**A**) Boxplots show that the RF algorithm had less sample residual than XGB, SVM, and RF. (**B**) Reverse cumulative distribution of sample residual indicated RF algorithm least residual. (**C**) Barplot highlights CXCL8 and TMSB10 in the RF algorithm as the most important genes for BA. (**D**) The chromosomal locations of CXCL8 and TMSB10. (**E**) The Drug-Gene Interaction Database filtered out the potential drugs targeting TMSB10 and CXCL8. (**F**) qRT-PCR confirmed that TMSB10 mRNA was expressed at high levels in BA liver samples. (**G**) Immunohistochemistry demonstrates the high expression of TMSB10 protein in BA liver samples.

Based on the 298 genes in the turquoise module, we applied four machine-learning algorithms (XGB, SVM, RF, and GLM) to establish models and evaluate their performance. The RF model with the least sample residual was selected (Figures 4A,B). Subsequently, the RF model identified *CXCL8* and *TMSB10* as the top important genes related to BA (Figure 4C). The chromosomal locations of CXCL8 and TMSB10 are shown in Figure 4D. We further screened potential drugs targeting *TMSB10* and *CXCL8* using the Drug-Gene Interaction database (DGIdb) (Figure 4E) (17). Previous studies have shown that CXCL8 is upregulated in the liver of patients with BA and could serve as a sensitive diagnostic and prognostic biomarker (18, 19). Our qRT-PCR and IHC experiments also indicated that the liver of patients with BA expressed higher *TMSB10* than patients with nBA at both mRNA and protein levels (Figures 4F,G).

### Validation of diagnostic values of the hub genes

**Figure 5:**
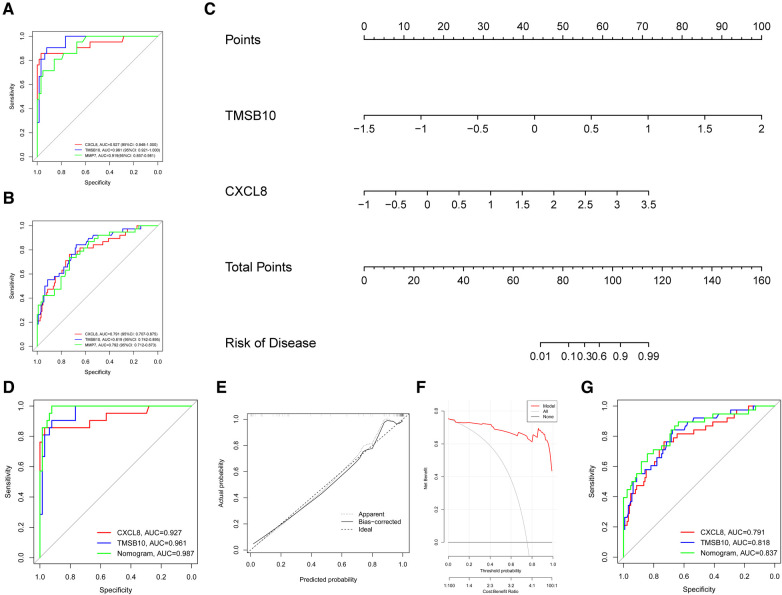
The diagnostic values of the hub genes. (**A**) The AUC of the ROC curve for hub genes *TMSB10* and *CXCL8* in the training group (GSE46995). (**B**) The AUC of the ROC curve for hub genes *TMSB10* and *CXCL8* in the testing group. (**C**) The nomogram combining hub genes *TMSB10* and *CXCL8* was built for better BA clinical diagnosis. (**D)** The AUC of the ROC for the nomogram in the training group. (**E**) The calibration curve for the nomogram exhibits consistency between the predicted and observed probabilities in the training groups. (**F**) The DCA curve shows the benefits acquired from the nomogram. (**G**) The AUC of the ROC for the nomogram in the testing group.

The ROC curve analysis revealed that *TMSB10* (AUC = 0.961, 95%CI: 0.921–1.000) and *CXCL8* (AUC = 0.927, 95%CI: 0.848–1.000) had at least similar diagnostic efficacy than clinical diagnostic biomarker *MMP7* (AUC = 0.919, 95%CI: 0.857–0.981) (Figure 5A). This diagnostic performance was similarly confirmed in the testing group, where *TMSB10* and *CXCL8* were found to be comparable to or better than *MMP7* (Figure 5B).

We constructed a nomogram based on biomarkers genes *CXCL8* and *TMSB10* in the training group for better BA clinical diagnosis (Figure 5C)**.** The ROC curve of the nomogram displayed an AUC of 0.987 in the training group (Figure 5D), indicating its strong ability to distinguish patients with BA from nBA. Calibration curves demonstrated outstanding consistency between the predicted and observed probabilities (Figure 5E). The C-index was 0.987. DCA indicated that patients with BA could benefit from the nomogram model (Figure 5F). Notably, the AUC of the ROC curve reached 0.837 in the testing group, indicating that it could reliably diagnose BA in various populations (Figure 5G).

### Investigation of immunological characteristics related to Ba

CIBERSORT analysis was performed to investigate the proportions of 22 immunocyte subtypes for each patient with BA in the training group (Supplementary Figure S1A). Our findings revealed that patients with BA had significantly higher proportions of eosinophils and resting memory CD4 *T* cells but significantly lower proportions of Treg, CD8T, follicular helper T, and plasma cells than those with nBA (Supplementary Figure S1B).

## Discussion

Biliary atresia (BA) is a progressive fibroinflammatory biliary obstructive disease and is the predominant reason for pediatric liver transplants (1). Timely Kasai portoenterostomy could rapidly restore bile flow, slow the rapid disease course and prevent the need for liver transplants (20). However, the clinical features of BA often overlap with other infantile cholestasis diseases, making it challenging to make a definitive and early diagnosis (21). The discovery of new biomarkers is essential for achieving accurate diagnosis and improving patient outcomes.

In recent decades, RNA-seq technology has become an indispensable tool for understanding the structure and function of the genome, identifying genetic networks underpinning cellular, physiological, biochemical, and biological systems, and developing molecular biomarkers for disease detection (22, 23). RNA-seq may provide clues to identify new biomarkers for BA. Thus, based on the mRNA expression data from BA and nBA liver biopsy samples from the GEO database, we identified 3,273 DEGs. It was then crucial to filter out several valuable diagnostic biomarkers from the thousands of DEGs.

Our study used WGCNA to further identify the gene modules closely related to BA. WGCNA is a regulatory network algorithm that constructs gene co-expression modules based on scale-free topology (24, 25). Besides, it could locate co-expression modules related to clinical features and identify potential disease biomarkers (26). It has proven to be a robust tool for analyzing gene expression data and works better than other weighted or unweighted networks for building gene network structures (26, 27). In our study, WGNCA categorized the DEGs into four gene coexpression modules and confirmed that the turquoise module exhibited the closest association with BA. Furthermore, enrichment analysis confirmed that genes in the turquoise module were primarily associated with ECM. Meanwhile, an imbalance in ECM deposition and breakdown has been implicated in the pathogenesis and progression of BA (28). Thus, WGNCA effectively identified the key gene module related to BA.

Machine learning methods offer a powerful means of processing complex and large genomic datasets, which are challenges for traditional statistical algorithms (29, 30). These methods have been widely used to identify novel biomarkers for detecting disease and predicting treatment response and disease outcome (30). For instance, based on transcriptomic data, Fortino V et al. identified potential biomarkers to distinguish allergic and irritant contact dermatitis (31). Our study also used machine learning algorithms to isolate diagnostic biomarkers from the gene module related to BA, identifying two key genes, *CXCL8* and *TMSB10*. External and internal validations were performed to verify the diagnostic value and confirmed that *CXCL8* and *TMSB10* could accurately diagnose BA. Besides, we developed a nomogram that integrated *CXCL8* and *TMSB10,* which exhibited higher diagnostic performance than a single gene and exhibited potential for clinical translation. Liver biopsy is the indispensable procedure for acquiring the expression of *CXCL8* and *TMSB10*.

*TMSB10* is a small G-actin-binding protein that promotes depolymerization of intracellular F-actin networks (32, 33). Amarachintha SP et al. reported that normal and diseased cholangiocyte-like organoids with normal cell polarity had an apical expression for F-actin, while depolarized BA cholangiocyte-like organoids expressed F-actin apically and basolaterally (34), indicating that *TMSB10* may regulate BA cell polarity via F-actin. Studies have shown that normal cell polarity is essential for proper bile duct development and function, while the disordered apical-basal polarity seen in BA contributes to the disease (35, 36). This suggests the potential of *TMSB10* as a therapeutic target for BA.

Previous studies have investigated the relationship between CXCL8 and BA. Bessho K et al. reported that CXCL8 in the liver serves as a sensitive diagnostic biomarker, and perturbing the CXCL8-CXCR2 signature in the murine model could reduce the course of cholestasis and the risk of biliary obstruction, thereby increasing the overall survival (37). Leung DH et al. reported that serum CXCL8 significantly correlates with liver stiffness in BA and can predict poor clinical outcomes (38). Thus, CXCL8 may be a diagnostic and prognostic biomarker and therapeutic target for BA.

Our study validated that *CXCL8* and *TMSB10* can be high-value hepatic diagnostic biomarkers for BA using bioinformatics methods. Previous studies have confirmed the diagnostic value of *CXCL8* in serum. Further research is needed to confirm whether *TMSB10* can be a serum diagnostic biomarker. Besides, both *CXCL8* and *TMSB10* are potential therapeutic targets, with *CXCL8*'s therapeutic potential already supported by prior research. *TMSB10* represents a novel avenue for investigation, and further experiments will be crucial in unraveling its role in BA.

## Data Availability

The original contributions presented in the study are publicly available. This data can be found here: [link/accession number]. Gene Expression Omnibus database (https://www.ncbi.nlm.nih.gov/geo/), (GSE46995) and (GSE122340). Original contributions presented in the study are included in the article/**Supplementary Material**, further inquiries can be directed to the corresponding author.

## Supplementary material

Supplementary Figure S1

The immunological characteristics related to BA (**A**) The relative percentages of 22 immunocyte subtypes for BA and nBA were determined in the training group using the CIBERSORT algorithm. (**B**) The mean relative proportions of 22 immunocyte subtypes between patients with BA and nBA.
