# Supplementary figures and images for: Identification of diagnostic biomarkers and potential therapeutic targets for biliary atresia via WGCNA and machine learning methods
Source: Front Pediatr. 2024 Jun 26;12:1339925. doi: 10.3389/fped.2024.1339925 (PMC11233743; doi:10.3389/fped.2024.1339925)

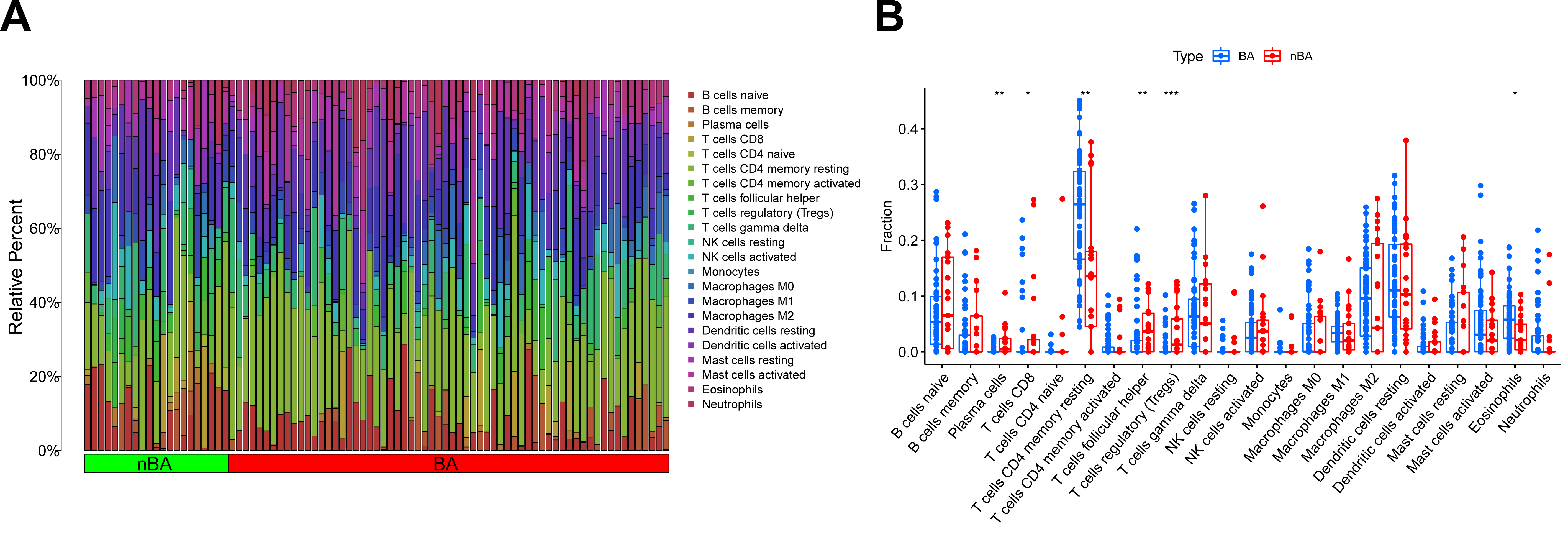

Supplement: Supplementary file 1 [file Image1.tif]
